# Spatial and temporal genetic variation in an exploited reef fish: The effects of exploitation on cohort genetic structure

**DOI:** 10.1111/eva.13198

**Published:** 2021-02-10

**Authors:** Zahra S. Taboun, Ryan P. Walter, Jennifer R. Ovenden, Daniel D. Heath

**Affiliations:** ^1^ Great Lakes Institute for Environmental Research (GLIER) University of Windsor Windsor Ontario Canada; ^2^ Department of Biological Science California State University, Fullerton Fullerton CA USA; ^3^ Molecular Fisheries Laboratory School of Biomedical Sciences University of Queensland Brisbane Queensland Australia; ^4^ Department of Integrative Biology University of Windsor Windsor Ontario Canada

**Keywords:** cohort, fishing pressure, genetic diversity, selection, spatial, temporal

## Abstract

Many coral reef fishes are fished, often resulting in detrimental genetic effects; however, reef fishes often show unpredictable patterns of genetic variation, which potentially mask the effects of fishing. Our goals were to characterize spatial and temporal genetic variation and determine the effects of fishing on an exploited reef fish, *Plectropomus leopardus*, Lacepède (the common coral trout). To determine population structure, we genotyped 417 Great Barrier Reef coral trout from four populations sampled in 2 years (1996 and 2004) at nine microsatellite loci. To test for exploitation effects, we additionally genotyped 869 individuals from a single cohort (ages 3–5) across eight different reefs, including fished and control populations. Genetic structure differed substantially in the two sampled years, with only 1 year exhibiting isolation by distance. Thus, genetic drift likely plays a role in shaping population genetic structure in this species. Although we found no loss of genetic diversity associated with exploitation, our relatedness patterns show that pulse fishing likely affects population genetics. Additionally, genetic structure in the cohort samples likely reflected spatial variation in recruitment contributing to genetic structure at the population level. Overall, we show that fishing does impact coral reef fishes, highlighting the importance of repeated widespread sampling to accurately characterize the genetic structure of reef fishes, as well as the power of analysing cohorts to avoid the impacts of recruitment‐related genetic swamping. The high temporal and spatial variability in genetic structure, combined with possible selection effects, will make conservation/management of reef fish species complex.

## 1 INTRODUCTION

Species from a wide range of habitats are experiencing the negative impacts of global climate change (Walther et al., 2002), specifically new selection pressures accompanying the environmental shifts and unpredictable climate patterns. This is problematic for species living in environments close to their physiological limits (Hoffman & Sgro, 2011), such as many in tropical habitats. For example, thermally sensitive corals have experienced increased frequencies of coral bleaching and extreme weather events, leading to decreased coral cover (Graham et al., 2006), resulting in indirect negative effects on other reef species. Additionally, many coral reef fishes are being directly impacted by increasing marine temperatures; since tropical species evolved in a relatively stable thermal environment, they typically have a narrow thermal tolerance range (Tewksbury et al., 2008), and slight increases in temperature and CO_2_ concentrations can reduce performance (Munday et al., 2012; Pratchett et al., 2017). Furthermore, it is likely that harvesting can reduce resilience of populations to the stress of climate change (Planque et al., 2010). For example, physiological states are related to vulnerability to exploitation (discussed by Lennox et al., 2017), and specifically, high‐performance aerobic scope phenotypes may be targeted by fishing (Duncan et al., 2019). Given the susceptibility of reef fishes to the combined stressors of climate change and fishing pressure, it is likely that those stressors also impact the genetic composition of individuals and populations.

Dispersal is an important coral reef fish life‐history trait resulting in population connectivity, an important factor in coral reef management plans (Almany et al., 2017; Planes et al., 2009; Roberts, 1997). Population genetic studies in marine species show typically high gene flow (Clarke et al., 2010; Unsworth et al., 2008; Waples, 1998), also true for coral reef fishes (Mora & Sale, 2002; Williamson et al., 2016). Although adult coral reef fish tend to be sedentary after settlement (Sale, 1991), early pelagic life stages are highly dispersive (Hepburn et al., 2009). However, genetic differentiation, despite high gene flow, has been reported in reef fishes; for example, Salas et al. (2010) found subtle genetic structure in *Stegastes partitus* (bicolor damselfish) within Costa Rica–Panama reefs. Additionally, Hogan et al. (2012) found evidence of local retention in the *S*.* partitus* (bicolor damselfish). Similarly, Taylor and Hellberg (2003) reported strong genetic differentiation in populations of *Elacatinus evelynae*, a cleaner goby, despite a larval pelagic duration of 21 days. Nevertheless, high levels of dispersal pose challenges for studying population differentiation within coral reef fishes, as new recruits cause a mixing of genotypes after each reproductive cycle (Hepburn et al., 2009). Given that many reef fishes remain on reefs post‐settlement (Sale, 1991), individual cohorts should display higher genetic structure. Thus, cohort‐level analyses allow genetic diversity and divergence of ‘populations’ to be estimated with reduced interference from gene flow, with the expectation that as the cohorts age, the effects of selection and drift should accumulate and become more pronounced.

Although reef fishes generally show limited genetic structure, they can exhibit unpredictable spatial and temporal patterns, perhaps reflecting stochastic larval dispersal and variable larval retention (Hepburn et al., 2009; Hogan et al., 2012). Some marine species exhibit genetic structure that does not adhere to population genetic models, a phenomenon referred to as chaotic genetic patchiness, first described by Johnson and Black (1982). This phenomenon has been observed in various species, including *Acanthaster planci* (Crown‐of‐thorns starfish; Nash et al., 1988), sea urchins (*Strongylocentrotus purpuratus*, see Edmands et al., 1996; *Echinometra mathaei*, see Watts et al., 1990), *Spisula ovalis* (a clam, see David et al., 1997), *S*.* partitus* (the bicolor damselfish; Hogan et al., 2010; Lacson & Morizot, 1991), among others. The variable and unpredictable spatial and temporal genetic structure often reported among marine fish populations highlights the importance of characterizing patterns of genetic structure over time to inform conservation and management actions with respect to a population's evolutionary potential and hence their susceptibility to environmental and exploitation stress.

Standing genetic variation is important in evaluating the evolutionary potential of populations (Barrett & Schluter, 2008). Populations that harbour high genetic diversity are typically better able to respond to selection because there is an increased chance that the favoured genotypes are present within the population. However, genetic variation can be reduced through exploitation (Allendorf et al., 2008), and this can negatively impact the evolutionary potential of harvested populations. For example, Hauser et al. (2002) used scale‐extracted DNA to show a significant decline in neutral genetic diversity in *Pagrus auratus* (New Zealand snapper) associated with aggressive harvesting. Losses of genetic diversity due to fishing pressure have also been reported for *Gadus morhua* (North Sea cod; Hutchinson et al., 2003), *Diplodus sargus* (white seabream; Pérez‐Ruzafa et al., 2006), *Hoplosthethus atlanticus* (orange roughy; Smith et al., 1991) and *Rexea solandri* (gemfish; Ovenden et al., 2020). Therefore, combined stressors, such as fishing pressure and climate change, may erode the evolutionary potential for populations to adapt, ultimately leading to population decline and possible extirpation.

While marine fish population genetic structure is expected to vary over space and time, individual species also present specific challenges for applications of conservation and management genetics. For example, exploited reef fishes that are experiencing climate change effects but also undergo sequential hermaphroditism may be the most vulnerable to the genetic consequences of harvesting because size‐selective fishing removes larger (and thus sex‐specific) individuals and potentially those with adaptive phenotypes (Duncan et al., 2019). The common coral trout, *Plectropomus leopardus*, is an iconic species found on Indo‐Pacific reefs and undergoes protogynous sequential hermaphroditism (Frisch et al., 2016). This species is the most economically important harvested fish on the Great Barrier Reef (GBR; Leigh et al., 2014). Additionally, *Plectropomus* species are a top predator, and thus, impacts on these species may have cascading effects (Graham et al., 2006). It is thus essential that we characterize the genetic structure of this species on the GBR and integrate these data into management and conservation plans (Palumbi, 2003). Although there have been published population genetic studies on *Plectropomus*, research on this species is limited (Frisch et al., 2016). Williamson et al. (2016) used microsatellite marker‐based parentage analyses to examine larval dispersal within *P*.* leopardus* and found dispersal can be quite extensive (median dispersal distances = 190 km). Such high dispersal potential is consistent with the lack of genetic structure reported in the same study (Williamson et al., 2016). Van Herwerden et al. (2009) found that some *P*.* leopardus* populations have meta‐population genetic structure and dynamics. Furthermore, patterns of recruitment can vary through space and time; for example, Russ et al. (1996) found one cohort of *P*.* leopardus* dominated the population on two marine reserves on the GBR for at least 3 years. The economic and ecological importance of this fish, coupled with its particular vulnerability, highlights the need to define patterns of gene flow, genetic structure, and genetic diversity, and how these may be affected by fishing pressure.

The genetic consequences of exploitation in the common coral trout remain unknown, despite its economic and ecological value. In this study, we first use microsatellite data from four unexploited populations to characterize spatial and temporal genetic variation in genetic diversity and structure. Secondly, we sampled a single cohort of fish over 3 years (ages 3–5) from eight reefs that differ in fishing pressure and tested for the effects of exploitation on genetic diversity and structure. We found chaotic spatial and temporal genetic structure makes repeated and widespread sampling critical when characterizing genetic structure in species such as *P*.* leopardus*. However, analysing individual cohorts reduces the effects of variable recruitment, making it possible to assess the effects of fishing pressure on genetic diversity and structure – a critical need for effective management and conservation. Our data also highlight possible changes in adaptive potential of populations that are facing pressure from exploitation on a backdrop of climate change.

## 2 MATERIALS AND METHODS

### 2.1 Sample collection

**FIGURE 1:**
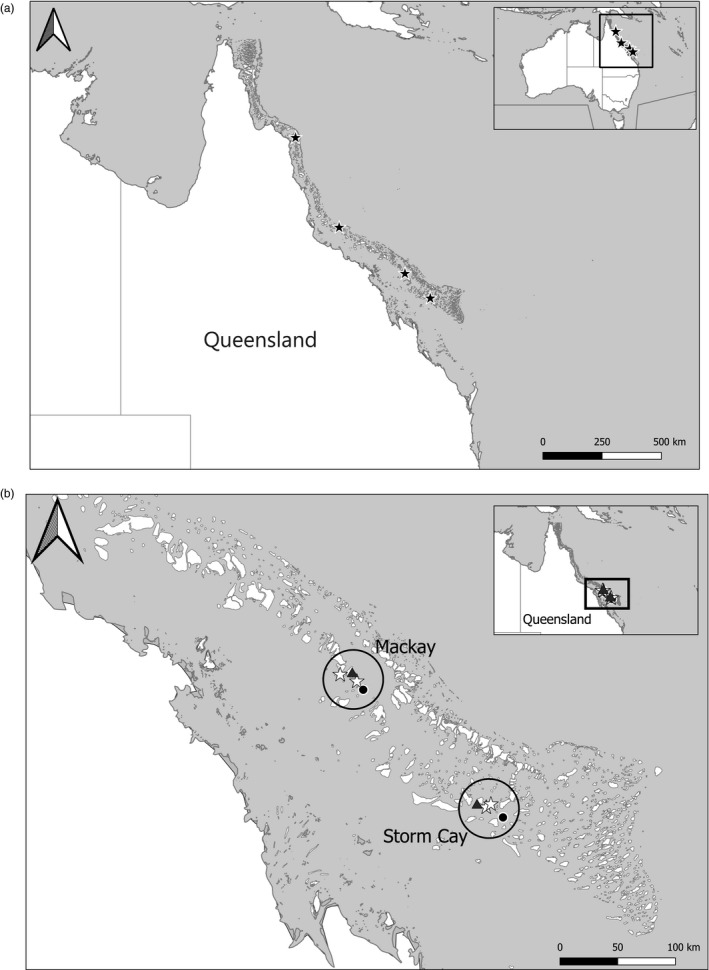
Maps showing the sampling sites for the coral trout used in this study. Panel (a) Map of the Australian Great Barrier Reef showing the sample sites for coral trout reefs included in spatial/temporal study (Table 1). Reef IDs from north to south: 14147, 18071, 20142 and 21132. Panel (b) Map of eight study sites within two spatial regions located on the Great Barrier Reef for coral trout populations included in cohort study (Table 1). Green reefs are represented by white stars, Manipulated reefs are represented by grey triangles, and Blue reefs are represented by black circles

The GBR, located on the eastern coast of Australia, is the world's largest coral reef ecosystem (Figure 1). As part of a large GBR project designed to test the effect of line fishing on GBR coral reef fishes, coral trout were collected from multiple reefs across much of the GBR (Mapstone et al., 2004). To reduce the introduction of potential sampling biases, times of peak spawning activity were avoided for the collection of the fish, as these periods could lead to unintentional sampling of spawning aggregations unassociated with true population genetic signals (Mapstone et al., 2004). Furthermore, sampling was standardized spatially and temporally on each reef (see Mapstone et al., 2004, for additional details on sampling). Therefore, it is likely that our samples are representative of the local population. The fish were collected using line fishing from reefs located in four regions: Lizard Island, Townsville, Mackay and Storm Cay (Figure 1a). Additionally, fish were sampled at eight reefs within two sub‐regions for a targeted fishing effects analysis; the reefs were categorized as Green (closed to fishing), Blue (open to fishing and subjected to pulse fishing in 1999) and Manipulated (closed to fishing but subjected to pulse fishing in 1999; Figure 1b). Pulse fishing included the maximum level of fishing possible without incentive for 12‐month periods by commercial and recreational fishers, as described in Mapstone et al. (2004). Reefs classified as ‘Green’ act as controls that provide measures of natural changes in genetic structure. Both the Blue and Manipulated reefs were treated with the same experimental pulse fishing schedule and allowed us to assess the fishing effects on populations compared to the Green controls. Otoliths were collected from all fish; however, age was determined only for the fish in the cohort genetic analysis (see below). The age of the fish was estimated as described in Mapstone et al. (2004) through otolith analyses. DNA was extracted from the otoliths following the protocol of Heath et al. (2007).

Two types of DNA analyses are included in this study, constituting two related, but separate analyses: (1) spatial/temporal genetic analyses and (2) cohort effects of fishing genetic analyses. The spatial/temporal samples include individuals obtained from four reefs, closed to fishing, sampled in 1996 and 2004 (Table 1 and Figure 1a). These samples allowed us to characterize spatial and temporal patterns of genetic variation at the population level. DNA samples included in the cohort study include individuals from a single cohort sampled over 3 years (1998–2000, ages 3–5) from eight reefs that differ in fishing pressures (see Table 1, Figure 1b and Table S1). The reefs in the cohort study are located in two regions, each region had four reefs: two Green, one Manipulated and one Blue (Figure 1b). Since long‐range and inter‐reef movements are relatively rare in coral trout (Matley et al., 2015; Sumpton et al., 2008), the cohort study allowed us to assess random and selection‐based genetic changes occurring in these populations over time. We also tested for fishing pressure effects with considerable power due to decreased interference from new recruits.

**TABLE 1:** Genetic sample information including sites, times and sample sizes for the common coral trout collected by line fishing on the Great Barrier Reef

Study	Spatial region	Reef	Status	Year	Age	Sample size
Spatial/temporal	Townsville	Glow Reef (18071)	Green	1996		59
				2004		60
	Lizard Island	South Direction Reef (14147)	Green	1996		42
				2004		32
	Mackay	U/N Reef (20142)	Green	1996		57
				2004		58
	Storm Cay	U/N Reef (21132)	Green	1996		58
				2004		51
Cohort	Storm Cay	U/N Reef (21132)	Green	1998	3	29
				1999	4	39
				2000	5	42
		Nancy Foster Reef (21131)	Green	1998	3	40
				1999	4	40
				2000	5	40
		U/N Reef (21133)	Manipulated	1998	3	27
				1999	4	37
				2000	5	18
		U/N Reef (21139)	Blue	1998	3	38
				1999	4	39
				2000	5	35
	Mackay	U/N Reef (20142)	Green	1998	3	40
				1999	4	29
				2000	5	40
		Robertson Reefs (No 3; 20137)	Green	1998	3	34
				1999	4	42
				2000	5	26
		Bax (20138)	Manipulated	1998	3	42
				1999	4	42
				2000	5	40
		Boulton (20146)	Blue	1998	3	33
				1999	4	39
				2000	5	38

Note

‘Age’ refers to the age of the cohort determined by otolith analysis.

Reef status refers to the fishing pressure status of the population: Green reefs are closed to fishing, Manipulated reefs were closed to fishing, but pulse fished in 1999, while Blue reefs were completely open to fishing and also pulse fished in 1999. Reef names are shown following identification number. Reefs labelled ‘U/N Reef’ are unnamed reefs. Age refers to the age of the fish as determined by Mapstone et al. (2004) through otolith analysis. Sample sizes varied per locus (ranging from 15–60).

### 2.2 Genotyping

Multiplex PCRs of 16 microsatellite loci were performed in 7.0 µl reactions (See Table S2 for loci) including replicates of eight individuals (as genotype controls) and negative controls. Each reaction included 3.5 µl of Microsatellite Type‐It Master Mix (Qiagen, Inc.), 0.8 µl of water, 0.7 µl of primer pool (with each primer having a final concentration of 0.2 µM) and 2 µl of sample DNA. Thermal cycling conditions were as follows: an initial denaturation of 95°C for 5 min, followed by 28 cycles of 3‐step cycling, including an initial denaturation of 30 s at 95°C, 90 s annealing of 57°C, and 30 s extension at 72°C, ending with a final extension of 30 min at 60°C. The PCR products were diluted 1:10 and then ligated with next‐Generation Sequencing (NGS) adapters and individual sample barcode sequences in a second (ligation) 20 µl PCR reaction containing: 20 mM Tris‐HCl (pH 8.8), 10 mM KCl, 10 mM (NH_4_)_2_SO_4_, 2 mM MgSO_4_, 0.1% Triton X‐100, 0.1 mg/ml bovine serum albumin, 200 µM of each dNTP, 200 nM of forward and reverse primers, 0.5 U of Taq polymerase (Bio Basic Canada Inc.) and 10 µl of diluted first‐round PCR product. Thermocycling conditions used for the ligation PCR were as follows: 94°C for 2 min, six cycles of 94°C for 30 s, 30 s at 60 and 72°C for 60 s, followed by 72°C for 5 min. The barcoded products were pooled, run on an agarose gel, and the bands were excised and cleaned using GenCatch Gel Extraction Kit (Epoch Life Science Inc.). The pooled amplicons were assessed on a 2100 Bioanalyzer (Agilent Technologies), diluted to 60 pM and sequenced using an Ion Torrent NGS personal genome machine (Thermo Fisher Scientific). We also sequenced negative controls from the first PCR.

We used QIIME (Caporaso et al., 2010) to demultiplex sequence data by individual sample code and to separate sequences based on primer sequences. We used NGS‐usat software (Roy et al., 2021) to genotype individuals. This R‐based software counts the number of microsatellite repeats within each sequence read and generates frequency‐based plots of read number versus repeat number. Automatic genotypes were generated, but alleles were visually verified and adjusted as necessary without knowledge of population assignment to reduce the potential for introducing biases. To determine the minimum sequencing depth required to reliably genotype individuals, we determined the number of reads at which heterozygosity estimates reached a plateau, which determined the minimum number of reads required for genotyping. We only included marker loci with five or more alleles across all samples and that were genotyped in 15 or more individuals per population for further analyses.

### 2.3 Statistical analyses

#### 2.3.1 Spatial/Temporal study

##### Population genetic diversity

Unless otherwise stated, statistical analyses were conducted using R Core Team (2019). We first characterized genetic diversity within populations by calculating the number of alleles, observed heterozygosity, expected heterozygosity and Hardy–Weinberg Equilibrium (HWE) deviations using R package ‘diveRsity’ (Keenan et al., 2013). To verify that each locus was independent, we tested for linkage disequilibrium for locus pairs in each population and year using ‘genepop’ v1.1.7 R package (Rousset, 2008). We assessed HWE and linkage‐equilibrium deviations after Bonferroni correction.

We used an individual‐level test to determine if *P*.* leopardus* populations on the GBR differ spatially and temporally in genetic diversity. To eliminate potential biases associated with missing genotypes, we used individuals genotyped successfully at all loci, (*n* = 258 with population sample sizes ranging from 18 to 46). We calculated individual heterozygosity, generated the mean heterozygosity across all genotyped loci and used a linear model with effects for population (*N* = 4) and year (1996 and 2004), and the interaction of the two terms using the ‘lme4’ v1.1–21 R package (Bates et al., 2015). We first tested for the interaction term by comparing a reduced model without the term to the full model. We then used a second linear model with effects for population and year. We then tested for evidence of spatial and temporal effects by comparing the full model to a reduced one without the ‘population’ or ‘year’ term, respectively (details on models are available under Supporting information). To test for temporal changes within reefs, we performed exact tests of population differentiation within reefs across the two time periods using contingency tables of allele frequencies implemented in the ‘genepop’ v1.1.7 R package (Rousset, 2008).

##### Population genetic divergence

We estimated global *F*_ST_ to quantify differentiation among subpopulations for both sample years separately using an analysis of molecular variance (AMOVA) with significance tested over 9999 permutations implemented in GenoDive v.3.04 (Meirmans, 2020). We also used GenoDive v.3.04 (Meirmans, 2020) to estimate pairwise *F*_ST_ for each population pair within each sampling year. Because estimates of *F*_ST_ can be relatively insensitive to subtle genetic structure, we also performed pairwise exact tests of population differentiation using the ‘genepop’ v1.1.7 R package (Rousset, 2008) for populations within each sampling year. Finally, to determine whether isolation was a function of distance among reefs, we tested for isolation by distance (IBD) using the ‘adegenet’ package in R (Jombart, 2008), which creates matrices of both pairwise genetic (Reynold's distance; Reynolds et al., 1983) and geographic (Euclidean distance) distance based on genotypes and reef coordinates and performs a Mantel test.

#### 2.3.2 Cohort study

##### Population genetic diversity

To assess the impacts of fishing pressure on genetic diversity in the cohort study populations, we used the R package ‘diveRsity’ (Keenan et al., 2013) to first obtain measures of diversity (heterozygosity and allelic richness). To eliminate potential biases caused by missing genotypes, we only used individuals that were successfully genotyped for all loci, (*n* = 684, with population sample sizes ranging from 14–39) to estimate allelic richness. To test for the effects of spatial region on genetic diversity, we used a linear mixed effects model in the ‘lme4’ v1.1–21 R package (Bates et al., 2015) with random effects for locus and fixed effects for spatial region, fishing pressure status, and cohort age. We used likelihood ratio tests to compare the full model to a reduced model to test for the significance of terms of interest. We used a second linear mixed effects model to assess interaction effects; this model included fixed effects for fishing pressure status, cohort age plus the interaction between cohort age and fishing pressure status, and random effects for reef and locus. We then used a likelihood ratio test as described above, and, where effects were significant, we conducted pairwise tests to determine the factor driving significance using the ‘lsmeans’ v2.30–0 R package (Lenth, 2016).

Furthermore, the effects of genetic drift should increase as the cohort ages and experiences cumulative losses. Since this process will drive changes in allele frequencies over time, we used an exact contingency‐table test in the ‘genepop’ v1.1.7 R package (Rousset, 2008), to test for changes in allele frequency distributions as the cohorts aged.

##### Population genetic divergence

As a cohort ages, and cohort size declines, genetic drift will increase and should be reflected in greater population genetic divergence. We predicted that populations that experience fishing pressure would undergo even faster divergence relative to our ‘Green’ reef controls due to greater cohort losses, leading to elevated genetic change. To detect spatial variation in cohort composition and to determine if patterns of genetic structure varied as the cohort aged, we first estimated Global *F*_ST_ for all three sampled cohort ages using GenoDive v3.04 (Meirmans, 2020) using an AMOVA with 9999 permutations. We also calculated per locus *F*_ST_ values for all pairwise comparisons among reefs within each spatial region as well as across spatial regions for each year using GenoDive v3.04 (Meirmans, 2020). These locus‐specific *F*_ST_ values represent independent measures of pairwise divergence. To determine if patterns of genetic divergence differed between reefs differing in fishing pressure status, we grouped the pairwise comparisons based on the fishing pressure status of the two reefs compared (e.g. Blue/Green, Green/Green, etc.). We then used a linear mixed effects model with fixed effects of comparison type, cohort age, and the relative location of the reefs being compared (i.e. within or across spatial region), and locus as a random effect. We used a second linear mixed effects model to test for interaction effects with fixed effects for the type of comparison, cohort age, and type‐by‐age interactions, and random effects for locus. We then used likelihood ratio tests to assess significance of terms of interest as described above. For effects that were significant based on the results from the linear mixed effects model, we used the ‘lsmeans’ v2.30–0 R package (Lenth, 2016) to conduct pairwise tests.

#### 2.3.3 Pairwise relatedness

As individuals age within a cohort, the mean relatedness among individuals is expected to increase if selection acts to favour certain families (Hogan et al., 2010). To determine if population relatedness was changing as the cohort aged, we used the ‘Demerelate’ v0.9–3 R package (Kraemer & Gerlach, 2017) to estimate pairwise relatedness among individuals within a population for each of the three age classes within the cohort. Demerelate uses a jackknife method to estimate the relatedness across random samples to assess neutral expectations and to aid in the selection of the most suitable estimator choice – we used the relatedness estimator of Queller and Goodnight (1989). Furthermore, Demerelate also provides estimates of the number of siblings and half siblings present in a population sample. Because we expected the cohort to experience selection as it aged, we expected that empirical data will contain more siblings and half siblings than the simulated datasets. To eliminate potential biases caused by missing genotypes, we only used individuals that were successfully genotyped for all loci, (*n* = 684, with population sample sizes ranging from 14 to 39).

We used two linear mixed effects models implemented in the ‘lme4’ R package (Bates et al., 2015) to investigate the impacts of fishing pressure and age on relatedness. The first model included reef as a random effect and fixed effects for fishing pressure status, cohort age and spatial region. The second model included fixed effects for spatial region, fishing pressure status, cohort age, and age by fishing pressure status interactions and reef as a random. We then tested the significance of terms of interest using a likelihood ratio test and used the ‘lsmeans’ v2.30–0 R package (Lenth, 2016) to conduct pairwise tests for effects that were significant based on the results from the global tests.

## 3 RESULTS

In total, we genotyped 1286 individuals, with 417 from the spatial/temporal study and 869 from the cohort study. Statistical analyses were carried out using nine microsatellite loci for the spatial/temporal study and eight loci for the cohort study. Seven loci were removed from both studies due to low number of alleles (PL05, PL07, PL11, PL13, PL14, PL15 and Pma191). PL09 was removed from the cohort study due to low genotyping success in one of the eight populations.

### 3.1 Spatial/temporal study

#### 3.1.1 Population genetic diversity

We found that 14 of 72 tests for HWE deviations were significant (Table S3), with all but one locus (Ple002) showing significant deviations in at least one population. Interestingly, six of the 14 significant deviations were in Reef 21132 in 2004, but in 1996, only one significant departure was detected suggestive of high levels of genetic drift (or selection). Additionally, 2 of 288 locus pairs showed linkage disequilibrium; however, they were not consistent across sampling sites or periods, indicating physical linkage of our markers is unlikely (Table S4).

**FIGURE 2:**
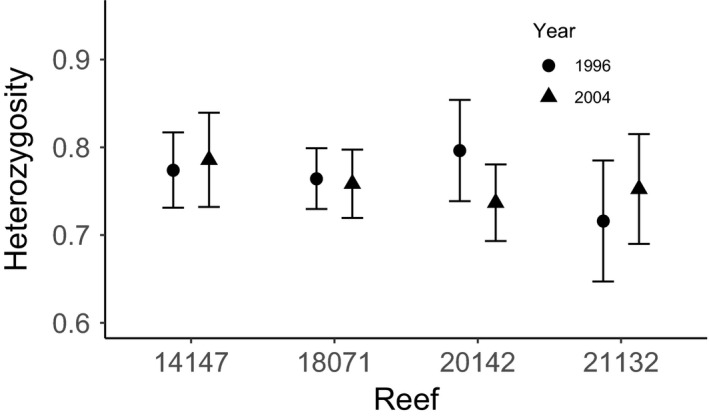
Mean individual heterozygosity (± 95% confidence intervals) for coral trout populations sampled at four reefs on the Great Barrier Reef for two sampling years (spatial/temporal study). ‘Population’ refers to the Reef ID assigned by the GBRMPA, see Table 1. Likelihood ratio tests showed no significant effects for year, population or year‐by‐population interactions

We measured individual‐level heterozygosity for the four populations in each year (Figure 2). Our likelihood ratio tests showed neither spatial (population effects: 0.46) nor temporal (year effects: *p* = 0.74) effects were significant for heterozygosity among the four populations included in the study. We also found that population‐by‐year interactions were not significant (*p* = 0.34) However, exact tests of allele frequency distribution showed that Reef 18071 (χ^2 ^= 38.25, df = 18, *p* = 0.0036) and Reef 21132 (χ^2^=39.5, df = 18, *p* = 0.0024) exhibited significant temporal change from 1996 to 2004.

#### 3.1.2 Population genetic divergence

We found evidence for both spatial and temporal variation in genetic structure; however, the patterns were not consistent (Table 2). Global *F*_ST_ values were 0.001 (*p* = 0.183) in 1996 and 0.004 (*p* = 0.009) in 2004. Although divergence in 1996 is low and not significant, there were some interesting patterns in the pairwise tests of genetic differentiation. In 1996, the pairwise *F*_ST_ values for the six population pairs ranged from −0.004 (*p* = 0.956) to 0.004 (*p* = 0.035, see Table S5 for all *p* values) and one pairwise *F*_ST_ value between two adjacent reefs (Reefs 20142 and 18071) significantly differed from zero. Additionally, the exact test of allele frequency distributions showed significant differentiation between two adjacent reefs (14147 and 18071), but not in any other comparisons (Table 2). These results are consistent with chaotic genetic patchiness (Table 2). In 2004, the pattern was different, with higher overall pairwise *F*_ST_ values ranging from −0.001 (*p* = 0.667) to 0.008 (*p* = 0.016), with three population pairs showing significant *F*_ST_ values and one pair that approached significance (Table 2). Furthermore, exact tests of allele frequency distribution differences among populations showed significant differentiation between three pairs of reefs, but not between the two reefs that differed in 1996 (Table 2).

**TABLE 2:** Pairwise *F*_ST_ values (±95% confidence intervals) (below the diagonal) and *p* values for exact test of population differentiation (above diagonal) for four coral trout populations located on the Great Barrier Reef in two sampling years (spatial/temporal study)

	Reef	14147	18071	20142	21132
1996	14147	—	**0.0004**	0.74	0.80
	18071	0.003 (±0.004)	—	0.15	0.053
	20142	0.001 (±0.005)	**0.004** (±0.006)	—	0.95
	21132	−0.004 (±0.001)	0.000 (±0.003)	−0.001 (±0.005)	—
2004	14147	—	0.27	0.20	**0.004**
	18071	**0.005** (±0.005)	—	**0.003**	**0.0003**
	20142	0.003 (±0.014)	*0*.*003* (±0.005)	—	**0.046**
	21132	**0.008** (±0.012)	**0.006** (±0.006)	−0.001 (±0.003)	—

Reefs are identified by numbers assigned by GBRMPA, see Table 1. Bolded *F*_ST_ values and bolded *p* values obtained from exact test are significant (*p* < 0.05), and italicized values approach significance (*p* < 0.10). See Table S5 for *p* values for *F*_ST_ tests.

Isolation by distance analyses showed that genetic divergence observed in 1996 was not a function of distance among the reefs 1996 (*p* = 0.748; *r* ^2^ = 0.032); however, the opposite was true in 2004 (*p* = 0.047; *r* ^2 ^= 0.77). These patterns of spatial population divergence are again, consistent with the chaotic genetic patchiness previously reported in coral reef fish populations (Hepburn et al., 2009; Hogan et al., 2010). It is likely that the ‘chaotic’ nature of the spatial and temporal variation we observed is at least partly due to our sampling of mixtures of genetically distinct cohorts, resulting from variable recruitment success.

### 3.2 Cohort study

#### 3.2.1 Population genetic diversity

**FIGURE 3:**
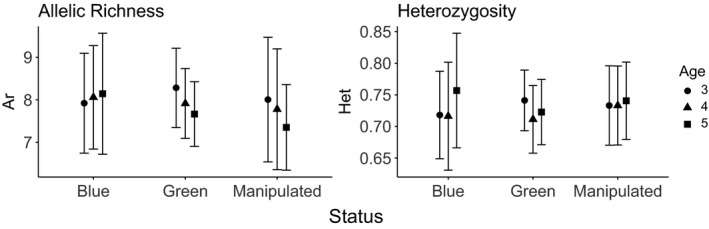
Mean genetic diversity (± 95% confidence intervals) estimates for the three years each cohort was sampled for coral trout from reefs differing in fishing pressure status (cohort study). ‘Status’ describes the fishing pressure status of the population (see Table 1; fishing pressure status refers to the fishing pressure status of the population: Green reefs are closed to fishing, Manipulated reefs were closed to fishing, but pulse fished in 1999, while Blue reefs were completely open to fishing and also pulse fished in 1999). Likelihood ratio tests showed overall age effects for allelic richness but not heterozygosity

**FIGURE 4:**
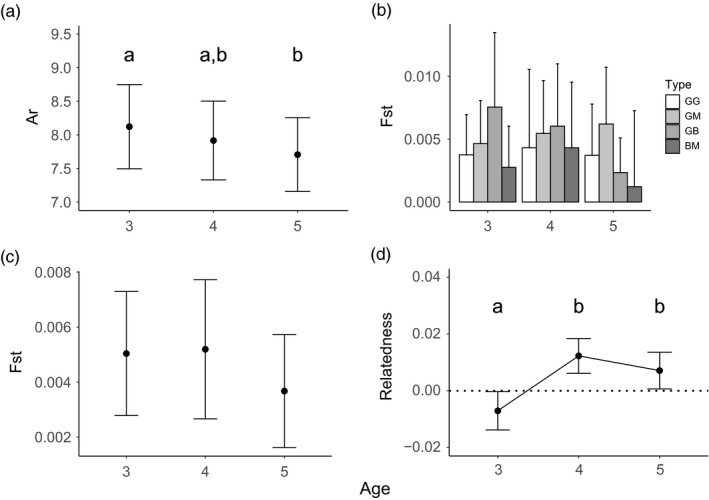
Mean diversity (± 95% confidence intervals) measures for three ages sampled across all eight populations of coral trout in the cohort study. (a): allelic richness estimates, showing a general decline in diversity as the fish aged. (b): Mean pairwise *F*_ST_ values for coral trout cohorts as they aged at sites differing in fishing pressure status. ‘Type’ refers to the type of comparison being made. GG is the control comparison between two Green reefs, GM refers to comparisons between Green and Manipulated reefs, GB refers to comparisons between Green and Blue Reefs, and BM refers to comparisons between Blue and Manipulated reefs. (c): pairwise *F*_ST_ values as the cohort aged. Likelihood ratio tests showed no significant changes. (d): pairwise relatedness, showing significant variation in relatedness as the cohorts aged

To determine the effects of cohort age and fishing pressure on genetic diversity, we measured allelic richness and heterozygosity in the cohorts across the three sampling years (Figure 3 and Table S6). Because we found no spatial region effects on allelic richness or heterozygosity, we dropped this term from all subsequent analyses (Table S7). Although we found no age effects on cohort heterozygosity, we found significant age (*p* = 0.0090) effects on allelic richness and age‐by‐status interaction (*p* = 0.093) and status (*p* = 0.083) effects that approached significance. As expected, allelic richness declined with age (Figure 4a, Table S7). To determine if fished reefs were more likely to undergo genetic change, we performed exact tests of allele frequency distribution change and found that the cohort underwent significant shifts in allele frequency distribution on 7 of the 8 reefs, with no apparent difference in the fished reefs (Table 3). Furthermore, there was variation in the timing of these shifts, depending on the reef examined.

**TABLE 3:** Uncorrected *p* values for tests of changes in allele frequency distributions for a cohort of coral trout across eight populations

Spatial region	Status	Reef	3/4 shift	4/5 shift	3/5 shift
Mackay	Green	20137	0.13	**0.038**	0.37
		20142	**0.0095**	0.80	0.64
	Manipulated	20138	**0.0075**	**<0.001**	**0.024**
	Blue	20146	0.29	0.45	0.64
Storm Cay	Green	21131	**<0.001**	0.96	**0.005**
		21132	**<0.001**	**0.0080**	0.22
	Manipulated	21133	**0.0037**	**0.032**	0.057
	Blue	21139	0.10	**0.004**	**0.002**

‘Spatial region’ refers to the region in which the reef is located, ‘Status’ refers to the fishing pressure status of the reef, and ‘Reef’ refers to the identification number assigned to the reef by the GBRMPA (Table 1). The numbers before ‘shift’ refer to the two age groups that were being compared. Bolded *p* values are significant.

#### 3.2.2 Population genetic divergence

We estimated Global *F*_ST_ for all three sampled cohort ages and found significant differentiation in all three age groups (Age 3: *F*_ST_ = 0.005, *p* = 0.001; Age 4 *F*_ST_ = 0.007, *p* < 0.001; Age 5: *F*_ST_ = 0.004, *p* = 0.002. We used pairwise *F*_ST_ estimates among reefs differing in fishing pressure status as the cohorts aged (Figure 4b, see Table S8) to test for effects on cohort genetic structure and found no significant effects for any of the terms tested (Table S9). Although we expected that the populations would show more divergence as the cohort aged, we found that *F*_ST_ values remained stable as the cohort aged from Age 3 to 5, independent of fishing pressure status (Figure 4b,c).

#### 3.2.3 Pairwise relatedness

**FIGURE 5:**
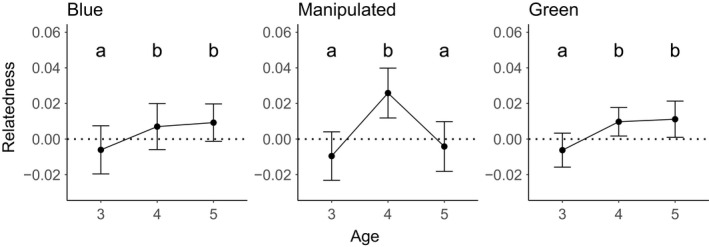
Mean pairwise relatedness (±95% confidence intervals) in coral trout population as the sampled cohort aged in reefs differing in fishing pressure status. The fishing pressure status of the reefs is indicated at the top of each graph (Blue, Manipulated, or Green). Letters indicate significant differences within fishing pressure status

Using pairwise relatedness estimates, we found that there were a greater number of full siblings (χ^2^ = 143.8, df = 1, *p* < 0.00001) in the entire cohort dataset than random expectation. Using two linear mixed models, we found that age effects and status‐by‐age interaction effects on pairwise relatedness were significant (Table S10). Overall, the average relatedness of the eight populations showed an increase from age 3 to 4 and remained stable from ages 4 to 5. (Figure 4d). In general, the Blue and Green reefs showed an increase in relatedness as the cohort aged, while Manipulated reefs showed an increase in relatedness from ages 3 to 4, followed by a decrease in relatedness from ages 4 to 5 (Figure 5).

## 4 DISCUSSION

We detected both temporal and spatial variation in the genetic structure of *P*.* leopardus* on the GBR. However, the patterns of divergence were indicative of chaotic genetic patchiness, with unpredictable variation over both space and time (Hepburn et al., 2009; Hogan et al., 2010; Johnson & Black, 1982). In general, genetic differentiation among reefs was low, indicating high levels of gene flow among populations. The combination of low overall divergence and high spatial and temporal variation is likely due to recurring recruitment waves of settling juveniles that led to changing mixtures of population genetic signatures (Hogan et al., 2012; Johnson & Black, 1982; Selkoe et al., 2006). In such a high gene flow environment, using a cohort approach to study population differentiation is valuable because the lack of adult dispersal decreases the chances of sampling an individual that immigrated into the ‘population’, but also allows us to assess changes that may be relevant at the population level. Our cohort study showed that selection acted on the cohort as it aged, but that there were few effects of line fishing on genetic diversity and population differentiation. However, intense pulse fishing pressure did drive changing patterns of pairwise relatedness as the cohort aged, indicating that different mechanisms of selection were likely acting to drive these changes.

Our spatial/temporal study showed that there was weak genetic structure among *P*. *leopardus* populations on the GBR and this genetic structure changes over time. Previous studies on common coral trout genetic structure on the GBR found no evidence of divergence (Williamson et al., 2016); however, the recruitment process in this species, and other coral reef fishes, likely leads to differences in genetic population structure depending on when and where samples are taken. For example, Russ et al. (1996) observed a particularly strong cohort of *P*.* leopardus* that was overrepresented in the population. Patterns of variable recruitment can be extremely important in driving chaotic genetic patchiness, likely due to a relatively small number of individuals contributing disproportionately to the next generation (Larson & Julian, 1999). Variable patterns of genetic structure are common in coral reef fishes; for example, one study showed that there was subtle genetic divergence in *S*.* partitus* (the bicolor damselfish) within the Mesoamerican barrier reef system (Hogan et al., 2010) despite other studies reporting no evidence of divergence (Ospina‐Guerrero et al., 2008). These inconsistencies in reported genetic structure within populations of coral reef fishes likely reflect high variation in recruitment and local retention (Hogan et al., 2012), which highlights the need for widespread monitoring repeated over time.

Interestingly, we saw elevated population differentiation and significant IBD in 2004, potentially indicating more restricted larval dispersal than in 1996. In general, larval dispersal is the main mechanism of connectivity in reef fish, as adults tend to remain on reefs once they have settled (Sale, 1991). However, climate change‐related stressors may impact patterns of connectivity among reefs. For example, Lo‐Yat et al. (2011) found that ocean warming reduced larval survival in tropical reef systems, and ocean acidification can further inflate larval mortality (Baumann et al., 2012) as well as alter settlement preferences (Devine et al., 2012). Additionally, Schunter et al. (2019) found that large variation in temperature, and hence pelagic larval duration, coincided with genetic patchiness patterns in *Tripterygion delaisi*, the black‐faced blenny. Finally, since habitat degradation can limit recruitment (McCormick et al., 2010), climate change coupled with habitat loss may play a key role in explaining some of the variation in coral reef fish genetic structure (O'Connor et al., 2007). Given that *P*.* leopardus* larvae can disperse over large distances (Williamson et al., 2016), it is likely that larval survival plays an important role in the connectivity of these populations, and in turn, their population dynamics and genetic structure.

As a cohort ages, genetic divergence among reefs should increase as selection and random genetic change occur, independent of fishing pressure. However, fishing pressure should increase those effects as fishing can be selective and reduced population size enhances random genetic change (Hoffman et al., 2011; Rodríguez‐Zárate et al., 2013). We know fishing pressure can contribute to selection (Barot et al., 2005; Haugen, & Vollestad, 2001; Morgan & Colbourne, 1999; Walsh et al., 2006) and would also tend to increase pairwise relatedness within a cohort. Our cohort analysis showed that changes in genetic structure and diversity occurred as the cohort aged. Overall, we found evidence of genetic change occurring both within and among reefs. As expected, overall allelic richness declined as the cohort aged, likely due to random genetic changes occurring as the size of the cohort inevitably decreased. Planes and Romans (2004) reported allozyme heterozygosity declining over time in a single cohort of *D*.* sargus* (white seabream). Although we expected a decrease in heterozygosity associated with cohort age, we did not find that effect, perhaps due to the lower sensitivity of heterozygosity as a genetic diversity measure relative to allelic richness. We would expect changes in allele frequency distribution to be the most sensitive measure of genetic change, and we found that allele frequency shifts tended to be common, showing that cohorts were likely to undergo genetic changes as they aged.

As a cohort ages, selection and immigration may occur and mean pairwise relatedness should change to reflect those cumulative changes (Hogan et al., 2010; Planes & Lenfant, 2002). For example, Planes and Lenfant (2002) reported significant fluctuations in mean pairwise relatedness in *D*.* sargus* (white seabream) cohorts, with the direction of change depending upon the specific cohort and the age of the individuals. We also found that the direction of change in pairwise relatedness varied with cohort age. Although our observed changes in relatedness may be explained by changing selective pressures (e.g., increased susceptibility of younger individuals to environmental stressors; Pörtner & Peck, 2010), it is also possible it may be due to immigration patterns. While increased relatedness as the cohorts age is consistent with selection reducing the number of families (Hogan et al., 2010), reduced relatedness as the cohort ages can most reasonably be explained by immigration of unrelated individuals (Planes & Lenfant, 2002). Although inter‐reef movements are thought to be rare for *P*.* leoaprdus* (Davies, 2000; Matley et al., 2015; Sumpton et al., 2008), they have been observed and are typically associated with spawning aggregations (Zeller, 1998), with older individuals more likely to make inter‐reef movements. Given that the mean age of maturity is 2.5 years in this species (Ferreira, 1995), we would be more likely to observe inter‐reef movements in the older individuals in our study, as they are more likely to be sexually mature. Furthermore, males are more likely to move to join spawning aggregations (Zeller, 1998), thus straying is more likely in older (male) individuals of this protogynous species. Finally, the reefs included in the cohort study are in close proximity, making inter‐reef movements at least possible, if perhaps still unlikely. Hence, spawning aggregation‐related dispersal of older individuals can provide an explanation of our observed pattern of change in relatedness, where increases in relatedness only occurred in the younger cohort ages. Furthermore, the population size of the older aged fish in a cohort is lower, and they are thus more likely to have mean relatedness affected by even rare immigration.

We found no evidence for losses in genetic diversity associated with exploitation among the coral trout at the eight reefs we sampled. There have been reports of harvested populations being resilient to losses in genetic diversity; for example, microsatellite analyses showed that high fishing pressure on a population of *Illex argentines* (squid) resulted in no significant loss of genetic diversity (Adcock et al., 1999). Additionally, we predicted that cohorts on fished reefs would be more likely to undergo shifts in allele frequencies; however, we found that seven of the eight reefs underwent significant shifts in allele frequency distribution with no apparent bias towards fished (Blue) reefs. This may be due to underlying differences among the populations; for example, habitat differences may lead to variation that masks the effects of fishing pressure (Ludwig et al., 1993; Mapstone et al., 2004; Payet et al., 2020). Additionally, Payet et al. (2020) found that *P*.* leopardus* populations on the GBR taken from fished reefs had smaller body sizes than those from protected reefs; however, individuals from populations from the Coral Sea (which experience minimal fishing pressure) were also smaller than those from protected areas on the GBR, likely indicating that other underlying processes are responsible for driving the reported size differences. Furthermore, inconsistencies in the population response to fishing pressure have previously been reported in this species. For example, *P*.* leopardus* populations in Western Australia experienced no declines in abundance, despite being open to fishing, while populations in protected areas declined in abundance, indicating some level of resilience to fishing pressure as well as variability in population mortality schedules (Mclean et al., 2011).

Evolutionary processes contribute to effective conservation of fishes (Harrison et al., 2012; Rieman & Allendorf, 2001) as both selection and drift can act to reduce the diversity of populations, while gene flow may increase diversity with important consequences for the management of populations in response to stressors such as fishing pressure (Allendorf et al., 2008) and climate change (Planque et al., 2010). The dispersal patterns of reef fishes are extremely important in designing marine reserves to ensure long term genetic replenishment from protected reefs to depleted (fished) reefs (Almany et al., 2017; Planes et al., 2009). For example, Harrison et al. (2012) showed that larval dispersal from protected reefs to fished reefs was substantial in *Plectropomus maculatas* populations on the GBR. Furthermore, Williamson et al. (2016) estimated the median dispersal distance of larval *P*.* leopardus* to be 190 km, which is greater than the distance between all the reefs included this study. Therefore, it is likely that Blue reefs experience an influx of larvae from the protected reefs included in this study, as well as from populations that were not sampled. Depending on the nature of the larvae that contribute to a single cohort, low relatedness at younger ages may reflect diverse larval source populations (such as from marine protected areas). Furthermore, the significant, but low, global *F*_ST_ values at each cohort age indicate that although spatial variation in recruitment likely contributed to cohort divergence, there was still genetic high similarity across the sampled populations. Clearly gene flow via larval dispersal is critical for replenishing diversity in fished reefs that experience the added stressors brought on by exploitation, highlighting the value of marine reserves in coral reef environments.

Fishing pressure has been shown to target specific families in a population, while others tend to avoid fishing mortality, thus inflating relatedness as populations are fished. For example, Saillant et al. (2003) found that *Lutjanus campechanus* (red snapper) in the Gulf of Mexico caught as by‐catch in fishing trawls were more related to each other than chance alone would predict, indicating that certain families are more susceptible to exploitation. In our study, both the Green and Blue (heavily fished) reefs experienced an increase in relatedness from ages 3 to 4, but not ages 3 to 5. Given that the protected and fished reefs showed similar patterns, it is unlikely to be selective effects related to fishing pressure. The lack of an effect on Blue reefs may be explained by the ongoing fishing pressure resulting in population adaptation to fishing‐related selection through social learning, which is thought to play a role in evading capture (discussed by Leigh et al., 2014). However, a different pattern of pairwise relatedness was observed for Manipulated reefs – the much larger increase in relatedness from age 3 to 4 in the manipulated populations may reflect strong selection on naïve populations, buffered at age 5 by possible gene flow, perhaps facilitated by the disturbance associated with the pulse fishing. Furthermore, the individuals in the Manipulated populations may also adapt to fishing pressure through social learning and thus are less affected at older ages. This is consistent with underwater visual survey data that showed pulse fishing did have an impact at the population level for Manipulated reefs (Mapstone et al., 2004). The susceptibility to individuals being captured is likely an interplay between size and naivety, with younger individuals likely being more naïve to fishing pressure, making them more susceptible to being captured and older individuals being larger, and therefore, more likely to be captured. However, larger fish accumulate toxins that cause human health issues (Lewis & Holmes, 1993; Wong et al., 2005) and are therefore less valuable for fisheries, which may reduce selection pressure on older individuals. Furthermore, the stable relatedness between Ages 4 and 5 on the Blue and Green reefs may reflect not only reduced natural mortality (and hence scope for selection) as the fish age, but perhaps also increased inter‐reef movements decreasing relatedness. Because we detected pulse fishing pressure effects at the cohort level, it is likely that fishing pressure plays an important role in shaping genetic structure at the population level.

In general, coral reef fishes tend to be challenging for population genetic applications in conservation and management, given their larval dispersal and diverse life histories. However, reef fishes are experiencing multiple stressors, and it is critical that these iconic species are conserved for both economic and ecological purposes, and genetic analyses can provide valuable insights. In this study, we focused on a particularly problematic reef fish species – *P*.* leopardus* are top‐predators that are actively exploited both commercially and recreationally on the GBR. Here, we report spatial and temporal chaotic genetic structure, a pattern previously reported for other reef fishes, but not for *P*.* leopardus*. Using DNA from known‐aged fish, we tracked an individual cohort as it aged, allowing us to examine populations without the confounding effects of new recruits changing the genetic structure of the ‘populations’. Furthermore, we have shown that pulse fishing likely affects *P*.* leopardus*, likely through selection, and resilience to stressors is likely population‐specific, and may be unpredictable, as other studies on *P*.* leopardus* populations have shown (Mapstone et al., 2004; Mclean et al., 2011). Variation in genetic patterns and resilience to stressors needs to be incorporated into models allowing for the sustainable harvest of coral reef fishes.

## CONFLICT OF INTEREST

The authors report no conflict of interest associated with this work.

## Supporting information

Supplementary Material

## Data Availability

Data for this study are available at: https://doi.org/10.5061/dryad.sbcc2fr5g
