# Supplementary material for: Spatial and temporal genetic variation in an exploited reef fish: The effects of exploitation on cohort genetic structure
Source: Evol Appl. 2021 Feb 10;14(5):1286–300. doi: 10.1111/eva.13198 (PMC8127707; doi:10.1111/eva.13198)
Supplement: Supplementary file 1 — Supplementary Material [file EVA-14-1286-s001.docx]

**Supplementary Material for:** Spatial and temporal genetic variation in an exploited reef fish: the effects of exploitation on cohort genetic structure

**Table S1:** Matrix showing distance (in km) between reefs included in the cohort study.

|  | **21132** | **21131** | **20142** | **20137** | **21133** | **20138** | **21139** | **20146** |
| --- | --- | --- | --- | --- | --- | --- | --- | --- |
| **21132** | -- |  |  |  |  |  |  |  |
| **21131** | 4.91 | -- |  |  |  |  |  |  |
| **20142** | 157.98 | 159.11 | -- |  |  |  |  |  |
| **20137** | 172.72 | 174.00 | 15.82 | -- |  |  |  |  |
| **21133** | 8.25 | 12.17 | 151.00 | 165.58 | -- |  |  |  |
| **20138** | 165.53 | 166.60 | 7.78 | 10.66 | 158.6 | -- |  |  |
| **21139** | 17.03 | 15.57 | 174.62 | 189.46 | 25.00 | 182.12 | -- |  |
| **20146** | 148.87 | 150.05 | 9.21 | 24.21 | 141.86 | 16.96 | 165.54 | -- |

**Table S2**: List of primers for *P. leopardus* microsatellite loci included in multiplex PCR used to genotype individuals from the Great Barrier Reef.

| Locus | Primer Sequence | Repeat Motif | GenBank Accession | Reference |
| --- | --- | --- | --- | --- |
| Ple001 | F: CATCACTGATCACACTGCCTCC  R: AACCTTCACTACAGTTAATACCACAC | ACGC | KF992544 | Harrison et al., 2014 |
| Ple002 | F: TACTCGCAATTATAACACAGATCCAG  R: TTTGTCCAGCACTGTATTTATCTATC | AGAT | KF992545 | Harrison et al., 2014 |
| PL03 | F: AGCTGCCAATCACCTGGTC  R: CCCACAAACCTGCTGGTCAT | CA | GU724990 | Zhang et al., 2010 |
| PL05 | F: GTATTGATGATGGGCTTGTGCT  R: GGATGAGACGGCGAACAAGG | AGG | GU724992 | Zhang et al., 2010 |
| PL07 | F: TGGCATCTCTGAGGGAGGA  R: ACGTTGTGTTTCTGCAGCTC | AG | GU724994 | Zhang et al., 2010 |
| PL09 | F: CCACAAACCTGCTGGTCATAT  R: CAATCACCTGGTCCGAAGTCT | (TG)T(GTGC) | GU724995 | Zhang et al., 2010 |
| PL11 | F: CACTCATGCTGTCCCTTTTCTC  R: TGCGAGGACACAGAGCAAGAA | (TC)(TG) | GU724997 | Zhang et al., 2010 |
| PL13 | F: TTCAGGGCCAATTTCAGCAG  R: AGCCCAAAACGTCAGGTCGA | GA | GU724998 | Zhang et al., 2010 |
| PL14 | F: AACGTCAGGTCGAGGTGTAG  R: GGACGCTCTATTTCCTGCTTA | (TC)(GAA) | GU724999 | Zhang et al., 2010 |
| PL15 | F: GAAGAGTTGAAGGTCGGTGCA  R: TGGGGTGATATGTGAGCAAAAG | (TA)G(CA) | GU725000 | Zhang et al., 2010 |
| Pma012 | F: ATATGGCCATTATTGTGAGTTAGGTG  R: AAATCTTTAAACCTACCACTGATCCC | AATG | KF992548 | Harrison et al., 2014 |
| Pma288 | F: TTGTATGTAATTTCGCCATGTTTGAG  R: TGTTGTCCGGTCATATTAATTGAGAG | AAAG | KF992562 | Harrison et al., 2014 |
| Pma412 | F: AAAGTTAGCCATTTAAACACAGAGCC  R: TAGGTAGAGGTCACTGTTGCATTATC | ACAT | KF992563 | Harrison et al., 2014 |
| Pma191 | F: GCCTTCGGAAACAATCATTATTCATC  R: GGGAAATTAAGAAGTCTACATTGAAGC | AAAT | KF992561 | Harrison et al., 2014 |
| Pma097 | F: AGTGGGGCCATGTTTAACAACAGCA  R: ACGAGTTTTGTGAGATGGATGGGTGGA | ATCT | JN222546 | Harrison et al., 2012 |
| Pma106 | F: CAGGAGCCATTGAGACAGGGAGAGG  R: AGTGTTGGTGGTTTCGCTGATGCTT | GATA | JN222548 | Harrison et al., 2012 |

**Table S3:** Summary statistics for population-level genetic diversity included in the spatial/temporal study. “Reef” refers to the reef ID assigned by the GBRMPA. Ho refers to observed heterozygosity and He is expected heterozygosity. HWE P values are based on chi-square tests: We tested for significance after Bonferroni correction. Bolded p values are significant after correction.

| **Reef** | **Year** | **Locus** | **Number of individuals genotyped** | **Number of alleles** | **Ho** | **He** | **HWE P value** |
| --- | --- | --- | --- | --- | --- | --- | --- |
| 14147 | 1996 | PL03 | 36 | 25 | 0.86 | 0.94 | 0.023 |
|  |  | PL09 | 36 | 23 | 0.69 | 0.93 | 0.011 |
|  |  | Ple001 | 42 | 11 | 0.74 | 0.86 | 0.43 |
|  |  | Ple002 | 41 | 13 | 0.9 | 0.88 | 0.88 |
|  |  | Pma012 | 42 | 11 | 0.81 | 0.81 | 0.89 |
|  |  | Pma097 | 42 | 11 | 0.81 | 0.85 | 0.93 |
|  |  | Pma106 | 42 | 17 | 0.86 | 0.90 | 0.024 |
|  |  | Pma288 | 41 | 7 | 0.61 | 0.59 | 0.033 |
|  |  | Pma412 | 41 | 9 | 0.49 | 0.66 | **0.00020** |
|  | 2004 | PL03 | 29 | 20 | 0.90 | 0.90 | **< 0.00010** |
|  |  | PL09 | 29 | 20 | 0.90 | 0.93 | 0.0037 |
|  |  | Ple001 | 32 | 11 | 0.69 | 0.88 | 0.016 |
|  |  | Ple002 | 32 | 17 | 0.72 | 0.84 | 0.94 |
|  |  | Pma012 | 32 | 8 | 0.72 | 0.77 | 0.27 |
|  |  | Pma097 | 32 | 10 | 0.88 | 0.86 | 0.69 |
|  |  | Pma106 | 32 | 13 | 0.75 | 0.84 | 0.046 |
|  |  | Pma288 | 32 | 6 | 0.62 | 0.66 | 0.98 |
|  |  | Pma412 | 32 | 8 | 0.69 | 0.64 | 0.082 |
| 18071 | 1996 | PL03 | 59 | 24 | 0.88 | 0.93 | **< 0.00010** |
|  |  | PL09 | 59 | 28 | 0.81 | 0.91 | 1.0 |
|  |  | Ple001 | 58 | 11 | 0.62 | 0.84 | 0.15 |
|  |  | Ple002 | 59 | 15 | 0.81 | 0.84 | 0.90 |

**Table S3 continued**

|  |  | Pma012 | 59 | 11 | 0.90 | 0.83 | 0.25 |
| --- | --- | --- | --- | --- | --- | --- | --- |
|  |  | Pma097 | 59 | 11 | 0.83 | 0.85 | 0.66 |
|  |  | Pma106 | 59 | 14 | 0.85 | 0.86 | 0.92 |
|  |  | Pma288 | 59 | 6 | 0.49 | 0.48 | 0.95 |
|  |  | Pma412 | 59 | 8 | 0.68 | 0.58 | 0.99 |
|  | 2004 | PL03 | 52 | 25 | 0.90 | 0.93 | **< 0.00010** |
|  |  | PL09 | 51 | 21 | 0.69 | 0.91 | **< 0.00010** |
|  |  | Ple001 | 57 | 12 | 0.70 | 0.86 | **0.00090** |
|  |  | Ple002 | 60 | 15 | 0.8 | 0.85 | 1.0 |
|  |  | Pma012 | 60 | 11 | 0.9 | 0.84 | 0.96 |
|  |  | Pma097 | 60 | 12 | 0.87 | 0.84 | 0.35 |
|  |  | Pma106 | 60 | 16 | 0.82 | 0.87 | 0.92 |
|  |  | Pma288 | 60 | 7 | 0.58 | 0.61 | 0.22 |
|  |  | Pma412 | 60 | 9 | 0.55 | 0.58 | 1.0 |
| 20142 | 1996 | PL03 | 18 | 17 | 0.83 | 0.92 | 0.22 |
|  |  | PL09 | 53 | 27 | 0.87 | 0.92 | 0.68 |
|  |  | Ple001 | 57 | 11 | 0.79 | 0.86 | 0.12 |
|  |  | Ple002 | 56 | 15 | 0.71 | 0.83 | 0.62 |
|  |  | Pma012 | 57 | 12 | 0.81 | 0.85 | 0.92 |
|  |  | Pma097 | 54 | 14 | 0.91 | 0.88 | 0.063 |
|  |  | Pma106 | 48 | 15 | 0.79 | 0.87 | 0.15 |
|  |  | Pma288 | 53 | 8 | 0.51 | 0.57 | 0.83 |
|  |  | Pma412 | 55 | 9 | 0.75 | 0.63 | 0.0085 |
|  | 2004 | PL03 | 54 | 29 | 0.81 | 0.93 | 0.0093 |
|  |  | PL09 | 45 | 25 | 0.73 | 0.92 | 0.0064 |
|  |  | Ple001 | 51 | 10 | 0.73 | 0.84 | 0.070 |
|  |  | Ple002 | 52 | 13 | 0.74 | 0.85 | 0.41 |
|  |  | Pma012 | 55 | 10 | 0.73 | 0.81 | 0.15 |
|  |  | Pma097 | 58 | 14 | 0.92 | 0.86 | 0.50 |

**Table S3 continued**

|  |  | Pma106 | 54 | 15 | 0.89 | 0.88 | 0.87 |
| --- | --- | --- | --- | --- | --- | --- | --- |
|  |  | Pma288 | 54 | 6 | 0.35 | 0.49 | 0.36 |
|  |  | Pma412 | 53 | 8 | 0.57 | 0.64 | 0.99 |
| 21132 | 1996 | PL03 | 28 | 19 | 0.82 | 0.92 | 0.15 |
|  |  | PL09 | 49 | 23 | 0.88 | 0.93 | 0.77 |
|  |  | Ple001 | 49 | 11 | 0.53 | 0.87 | **< 0.00010** |
|  |  | Ple002 | 53 | 16 | 0.87 | 0.87 | 0.96 |
|  |  | Pma012 | 57 | 11 | 0.74 | 0.82 | 0.99 |
|  |  | Pma097 | 58 | 12 | 0.79 | 0.87 | 0.34 |
|  |  | Pma106 | 53 | 15 | 0.83 | 0.89 | 0.50 |
|  |  | Pma288 | 54 | 9 | 0.43 | 0.58 | **< 0.00010** |
|  |  | Pma412 | 54 | 7 | 0.54 | 0.58 | 0.13 |
|  | 2004 | PL03 | 43 | 26 | 0.91 | 0.94 | 0.0053 |
|  |  | PL09 | 31 | 20 | 0.68 | 0.89 | 0.22 |
|  |  | Ple001 | 39 | 11 | 0.59 | 0.84 | **0.00010** |
|  |  | Ple002 | 44 | 12 | 0.73 | 0.80 | 0.0053 |
|  |  | Pma012 | 43 | 11 | 0.70 | 0.87 | **0.00030** |
|  |  | Pma097 | 47 | 11 | 0.74 | 0.86 | **< 0.00010** |
|  |  | Pma106 | 44 | 13 | 0.73 | 0.86 | **< 0.00010** |
|  |  | Pma288 | 43 | 8 | 0.37 | 0.50 | **0.00020** |
|  |  | Pma412 | 37 | 10 | 0.59 | 0.94 | **< 0.00010** |

**Table S4:** P values resulting from tests of linkage between locus pairs (indicated by Locus 1 and Locus 2) for four coral trout populations in two sampling years. P values were assessed for significance after Bonferroni correction. Bolded p values are significant after correction.

|  |  | 20142 | | 14147 | | 18071 | | 21132 | |
| --- | --- | --- | --- | --- | --- | --- | --- | --- | --- |
| Locus 1 | Locus 2 | 1996 | 2004 | 1996 | 2004 | 1996 | 2004 | 1996 | 2004 |
| PL03 | PL09 | 1.0 | 0.17 | 0.14 | 1.0 | 0.15 | **<0.0001** | 1.0 | 1.0 |
| PL03 | Ple001 | 1.0 | 0.66 | 1.0 | 1.0 | **<0.0001** | 0.15 | -- | 0.62 |
| PL09 | Ple001 | 1.0 | 0.22 | 1.0 | 1.0 | 0.72 | 0.78 | 1.0 | 1.0 |
| PL03 | Ple002 | 1.0 | 1.0 | 1.0 | 0.42 | 0.031 | 0.095 | 1.0 | 1.0 |
| PL09 | Ple002 | 1.0 | 1.0 | 1.0 | 1.0 | 0.56 | 1.0 | 1.0 | 0.65 |
| Ple001 | Ple002 | 0.58 | 1.0 | 1.0 | 1.0 | 0.28 | 0.43 | 0.083 | 1.0 |
| PL03 | Pma012 | 1.0 | 0.75 | 0.26 | 1.0 | 1.0 | 0.19 | 1.0 | 1.0 |
| PL09 | Pma012 | 0.34 | 1.0 | 0.093 | 1.0 | 0.62 | 0.72 | 0.039 | 0.50 |
| Ple001 | Pma012 | 0.87 | 0.90 | 0.72 | 0.13 | 0.98 | 1.0 | 0.47 | 0.37 |
| Ple002 | Pma012 | 0.007 | 0.94 | 1.0 | 1.0 | 0.11 | 0.48 | 0.88 | 0.023 |
| PL03 | Pma097 | 1.0 | 1.0 | 0.16 | 1.0 | 0.43 | 1.0 | 1.0 | 1.0 |
| PL09 | Pma097 | 0.28 | 0.45 | 1.0 | 1.0 | 1.0 | 0.67 | 1.0 | 0.44 |
| Ple001 | Pma097 | 0.77 | 0.90 | 0.10 | 0.35 | 0.92 | 0.15 | 1.0 | 0.025 |
| Ple002 | Pma097 | 0.19 | 0.038 | 1.0 | 1.0 | 0.90 | 1.0 | 0.44 | 1.0 |
| Pma012 | Pma097 | 0.85 | 0.33 | 0.35 | 0.057 | 0.88 | 0.06 | 0.012 | 0.28 |
| PL03 | Pma106 | 1.0 | 0.32 | 1.0 | 1.0 | 1.0 | 1.0 | 1.0 | 0.094 |
| PL09 | Pma106 | 1.0 | 0.35 | 0.091 | 1.0 | 0.24 | 1.0 | 1.0 | 0.41 |
| Ple001 | Pma106 | 0.73 | 0.65 | 0.054 | 0.19 | 0.49 | 0.43 | 0.49 | 0.35 |
| Ple002 | Pma106 | 0.81 | 0.46 | 0.19 | 1.0 | 1.0 | 0.26 | 1.0 | 0.78 |
| Pma012 | Pma106 | 1.0 | 0.93 | 0.0070 | 0.077 | 0.73 | 1.0 | 0.75 | 0.14 |
| Pma097 | Pma106 | 0.28 | 0.86 | 1.0 | 0.37 | 0.82 | 0.26 | 0.27 | 0.72 |

**Table S4 continued**

| PL03 | Pma412 | 1.0 | 0.095 | 1.0 | 0.54 | 0.057 | 0.49 | 0.44 | 0.68 |
| --- | --- | --- | --- | --- | --- | --- | --- | --- | --- |
| PL09 | Pma412 | 0.88 | 0.064 | 0.35 | 1.0 | 0.70 | 0.62 | 0.55 | 0.38 |
| Ple001 | Pma412 | 0.70 | 0.51 | 0.41 | 0.66 | 0.43 | 0.81 | 0.41 | 0.029 |
| Ple002 | Pma412 | 0.14 | 0.92 | 0.071 | 0.55 | 0.83 | 0.40 | 0.74 | 0.034 |
| Pma012 | Pma412 | 0.88 | 0.74 | 0.74 | 0.31 | 0.013 | 0.010 | 0.18 | 0.95 |
| Pma097 | Pma412 | 0.74 | 0.61 | 0.24 | 0.86 | 0.30 | 0.82 | 0.15 | 0.095 |
| Pma106 | Pma412 | 0.45 | 0.77 | 0.61 | 0.32 | 0.15 | 0.19 | 0.86 | 0.60 |
| PL03 | Pma288 | 1.0 | 0.31 | 1.0 | 0.89 | 0.98 | 0.52 | 0.31 | 0.73 |
| PL09 | Pma288 | 0.48 | 0.83 | 0.38 | 0.49 | 0.10 | 0.89 | 0.56 | 0.57 |
| Ple001 | Pma288 | 0.47 | 0.68 | 0.79 | 0.28 | 0.58 | 0.004 | 0.89 | 0.77 |
| Ple002 | Pma288 | 0.95 | 0.090 | 0.79 | 0.036 | 0.28 | 0.62 | 0.70 | 0.46 |
| Pma012 | Pma288 | 0.38 | 1.0 | 0.93 | 0.66 | 0.84 | 0.23 | 0.52 | 0.97 |
| Pma097 | Pma288 | 0.16 | 0.99 | 0.57 | 0.012 | 0.39 | 0.96 | 0.82 | 0.89 |
| Pma106 | Pma288 | 0.97 | 0.54 | 0.85 | 0.65 | 0.041 | 0.97 | 0.93 | 0.080 |
| Pma412 | Pma288 | 0.33 | 0.73 | 0.22 | 0.49 | 0.40 | 0.22 | 0.96 | 0.11 |

**Table S5:** Pairwise F_ST_ values (below diagonal) and p values for F_ST_ estimates (above diagonal) for four coral trout populations located on the Great Barrier Reef in two sampling years (spatial/temporal study). Reefs are identified by numbers assigned by GBRMPA, see Table 1. Bolded p values are significant (p<0.05) and italicized values approach significance (p<0.10).

| 1996 | Reef | 14147 | 18071 | 20142 | 21132 |
| --- | --- | --- | --- | --- | --- |
|  | 14147 | — | *0.069* | 0.243 | 0.956 |
|  | 18071 | *0.003* | — | **0.035** | 0.537 |
|  | 20142 | 0.001 | **0.004** | — | 0.726 |
|  | 21132 | -0.004 | 0.000 | -0.001 | — |
|  |  |  |  |  |  |
| 2004 | Reef | 14147 | 18071 | 20142 | 21132 |
|  | 14147 | — | **0.049** | 0.149 | **0.016** |
|  | 18071 | **0.005** | — | *0.067* | **0.007** |
|  | 20142 | 0.003 | *0.003* | — | 0.667 |
|  | 21132 | **0.008** | **0.006** | -0.001 | — |

**Table S6:** Genetic diversity measures for coral trout cohorts at reefs differing in fishing pressure status across 3 years. Values shown represent means, and 95% confidence intervals are shown in brackets. Reef status refers to the management of the population: green control reefs are closed to fishing, green manipulated were closed to fishing, but pulse fished in 1999, while blue reefs are completely open to fishing and also pulse fished in 1999. “Reef” refers to Reef identification number assigned by GBRMPA. “Spatial region” refers to the region the reefs are located in. “Year” refers to the year in which sampling took place, while “Age” refers to the age of the individuals as determined by otolith analysis.

| Spatial region | Reef | Status | Year | Age | Het_Obs_ | Ar |
| --- | --- | --- | --- | --- | --- | --- |
| Storm Cay | 21132 | Green | 1998 | 3 | 0.74 (0.63-0.85) | 8.17 (6.48-9.85) |
|  |  |  | 1999 | 4 | 0.64 (0.54-0.74) | 7.57 (6.10-9.04) |
|  |  |  | 2000 | 5 | 0.68 (0.60-0.76) | 7.19 (5.96-8.40) |
|  | 21131 | Green | 1998 | 3 | 0.79 (0.73-0.85) | 8.49 (6.37-10.6) |
|  |  |  | 1999 | 4 | 0.71 (0.61-0.81) | 7.88 (6.41-9.36) |
|  |  |  | 2000 | 5 | 0.77 (0.68-0.86) | 7.86 (6.23-9.49) |
|  | 21133 | Manipulated | 1998 | 3 | 0.71 (0.64-0.78) | 7.63 (5.85-9.40) |
|  |  |  | 1999 | 4 | 0.75 (0.66-0.82) | 7.92 (5.96-9.88) |
|  |  |  | 2000 | 5 | 0.68 (0.61-0.75) | 7.20 (5.81-8.59) |
|  | 21139 | Blue | 1998 | 3 | 0.72 (0.64-0.80) | 8.04 (6.34-9.74) |
|  |  |  | 1999 | 4 | 0.68 (0.56-0.80) | 7.95 (6.15-9.74) |
|  |  |  | 2000 | 5 | 0.72 (0.58-0.86) | 8.90 (6.57-11.24) |
| Mackay | 20142 | Green | 1998 | 3 | 0.75 (0.65-0.85) | 8.29 (6.40-10.17) |
|  |  |  | 1999 | 4 | 0.72 (0.59-0.85) | 7.83 (6.17-9.49) |
|  |  |  | 2000 | 5 | 0.73 (0.63-0.83) | 8.13 (6.36-9.90) |
|  | 20137 | Green | 1998 | 3 | 0.68 (0.58-0.78) | 8.18 (6.36-10.03) |
|  |  |  | 1999 | 4 | 0.77 (0.69-0.85) | 8.38 (6.43-10.33) |
|  |  |  | 2000 | 5 | 0.71 (0.58-0.84) | 7.49 (6.11-8.86) |
|  | 20138 | Manipulated | 1998 | 3 | 0.75 (0.65-0.85) | 8.39 (6.26-10.5) |

**Table S6 continued**

|  |  |  | 1999 | 4 | 0.71 (0.61-0.81) | 7.64 (5.78-9.49) |
| --- | --- | --- | --- | --- | --- | --- |
|  |  |  | 2000 | 5 | 0.80 (0.73-0.87) | 7.51 (6.19-8.82) |
|  | 20146 | Blue | 1998 | 3 | 0.72 (0.62-0.82) | 7.80 (6.34-9.26) |
|  |  |  | 1999 | 4 | 0.76 (0.66-0.86) | 8.17 (6.71-9.64) |
|  |  |  | 2000 | 5 | 0.80 (0.70-0.90) | 8.35 (6.54-10.16) |

**Table S7:** Uncorrected p values obtained from likelihood ratio tests testing for effects of fishing pressure status on genetic diversity in a cohort across 8 populations on the Great Barrier Reef.

| Response | Model | Fixed | Random | Factor | χ^2^ | df | P value |
| --- | --- | --- | --- | --- | --- | --- | --- |
| Heterozygosity | 1 | Region  Age  Status | Locus | Region | 1.2 | 1 | 0.27 |
|  |  |  |  | Age | 1.77 | 2 | 0.41 |
|  |  |  |  | Status | 0.57 | 2 | 0.75 |
|  | 2 | Status  Age  Age/status interactions | Reef  Locus | Age/status interactions | 2.55 | 4 | 0.63 |
|  |  |  |  | Reef | 3.11 | 1 | *0.078* |
| Allelic richness | 1 | Region  Age  Status | Locus | Region | 3.10 | 1 | *0.078* |
|  |  |  |  | Age | 9.43 | 2 | **0.0090** |
|  |  |  |  | Status | 4.97 | 2 | *0.083* |
|  | 2 | Status  Age  Age/status interactions | Reef  Locus | Age/status interactions | 7.95 | 4 | *0.093* |
|  |  |  |  | Reef | 3.17 | 1 | 0.53 |

“Fixed” and “Random” refer to the designation of factors, while “Factor” refers to the specific factor tested. Bolded p values are significant, while italicized values approach significance.

**Table S8:** Pairwise F_ST_ values (below diagonal) and p values for F_ST_ estimates (above diagonal) for one cohort from eight populations of coral trout located on the Great Barrier Reef. Estimates were calculated using 8 microsatellite loci. Reefs are identified by numbers assigned by GBRMPA, see Table 1. Bolded p values are significant after Bonferroni correction.

| Age |  | **20137** | **20142** | **20138** | **20146** | **21131** | **21132** | **21133** | **21139** |
| --- | --- | --- | --- | --- | --- | --- | --- | --- | --- |
| 3 | **20137** | -- | 0.688 | 0.326 | 0.341 | 0.474 | 0.044 | 0.120 | 0.020 |
|  | **20142** | -0.002 | -- | 0.179 | 0.044 | 0.605 | 0.013 | 0.028 | **0.001** |
|  | **20138** | 0.001 | 0.002 | -- | 0.132 | 0.32 | 0.002 | 0.567 | 0.134 |
|  | **20146** | 0.001 | 0.006 | 0.003 | -- | 0.40 | 0.063 | 0.090 | 0.008 |
|  | **21131** | 0 | -0.001 | 0.001 | 0.001 | -- | 0.036 | 0.090 | 0.008 |
|  | **21132** | 0.008 | 0.009 | 0.012 | 0.006 | 0.004 | -- | 0.58 | 0.014 |
|  | **21133** | 0.005 | 0.008 | 0.006 | 0.006 | 0.005 | -0.001 | -- | 0.618 |
|  | **21139** | 0.009 | **0.014** | 0.003 | 0.010 | 0.008 | 0.009 | -0.001 | -- |
|  |  | **20137** | **20142** | **20138** | **20146** | **21131** | **21132** | **21133** | **21139** |
| 4 | **20137** | -- | 0.82 | 0.094 | 0.151 | 0.032 | 0.079 | 0.021 | 0.369 |
|  | **20142** | -0.003 | -- | 0.312 | 0.523 | 0.600 | 0.019 | 0.382 | 0.346 |
|  | **20138** | 0.004 | 0.001 | -- | 0.191 | 0.095 | **<0.001** | 0.007 | 0.034 |
|  | **20146** | 0.003 | 0 | 0.002 | -- | 0.034 | **<0.001** | 0.355 | 0.002 |
|  | **21131** | 0.006 | -0.001 | 0.004 | 0.006 | -- | **<0.001** | 0.784 | **<0.001** |
|  | **21132** | 0.004 | 0.009 | **0.017** | **0.015** | **0.019** | -- | **<0.001** | 0.007 |
|  | **21133** | 0.006 | 0.001 | 0.009 | 0.001 | -0.002 | **0.017** | -- | **<0.001** |
|  | **21139** | 0.001 | 0.001 | 0.006 | 0.011 | **0.020** | 0.010 | **0.018** | -- |
|  |  | **20137** | **20142** | **20138** | **20146** | **21131** | **21132** | **21133** | **21139** |
| 5 | **20137** | -- | 0.037 | 0.053 | 0.486 | 0.004 | 0.130 | **0.001** | 0.021 |
|  | **20142** | 0.008 | -- | 0.035 | 0.650 | 0.784 | 0.768 | 0.669 | 0.202 |
|  | **20138** | 0.006 | 0.006 | -- | 0.664 | 0.010 | 0.171 | 0.006 | **0.001** |
|  | **20146** | 0 | -0.001 | -0.001 | -- | 0.043 | 0.508 | 0.643 | 0.105 |

**Table S8 continued**

|  | **21131** | 0.012 | -0.002 | 0.007 | 0.005 | -- | 0.031 | 0.134 | 0.146 |
| --- | --- | --- | --- | --- | --- | --- | --- | --- | --- |
|  | **21132** | 0.005 | -0.003 | 0.003 | 0 | 0.006 | -- | 0.093 | 0.144 |
|  | **21133** | **0.021** | -0.002 | 0.014 | -0.002 | 0.005 | 0.007 | -- | 0.593 |
|  | **21139** | 0.009 | 0.003 | **0.013** | 0.004 | 0.003 | 0.004 | -0.001 | -- |

**Table S9**: Results from likelihood ratio test assessing for significance of effects on genetic divergence for a cohort across 8 populations.

| Model | Fixed | Random | Factor | χ^2^ | df | P value |
| --- | --- | --- | --- | --- | --- | --- |
| 1 | Type  Age  Location | Locus | Type | 2.61 | 3 | 0.46 |
|  |  |  | Age | 1.15 | 2 | 0.56 |
|  |  |  | Location | 0.96 | 1 | 0.33 |
| 2 | Type  Age  Type/Age interactions | Locus | Type/Age interactions | 3.42 | 6 | 0.75 |

“Fixed” and “Random” refer to the designation of factors, while “Factor” refers to the specific factor tested. Bolded p values are significant.

**Table S10:** Results from likelihood ratio test assessing for significance of effects on pairwise relatedness for a cohort across 8 populations.

| Model | Fixed | Random | Factor | χ^2^ | df | P value |
| --- | --- | --- | --- | --- | --- | --- |
| 1 | Status  Age  Region | Reef | Status | 0.029 | 2 | 0.99 |
|  |  |  | Age | 22.29 | 2 | **1.44e-5** |
|  |  |  | Region | 0.46 | 1 | 0.50 |
| 2 | Status  Age  Status/Age interactions  Region | Reef | Status/Age interactions | 12.2 | 4 | **0.016** |

“Fixed” and “Random” refer to the designation of factors, while “Factor” refers to the specific factor tested. Bolded p values are significant. Italicized values approach significance.

**REFERENCES**

Harrison, H. B., Williamson, D. H., Evans, R. D., Almany, G. R., Thorrold, S. R., Russ, G. R., ... & Berumen, M. L. (2012). Larval export from marine reserves and the recruitment benefit for fish and fisheries. *Current biology*, *22*(11), 1023-1028.

Harrison, H. B., Feldheim, K. A., Jones, G. P., Ma, K., Mansour, H., Perumal, S., ... & Berumen, M. L. (2014). Validation of microsatellite multiplexes for parentage analysis and species discrimination in two hybridizing species of coral reef fish (*Plectropomus* spp., Serranidae). *Ecology and Evolution*, *4*(11), 2046-2057.

Zhang, J., Liu, H., & Song, Y. (2010). Development and characterization of polymorphic microsatellite loci for a threatened reef fish *Plectropomus leopardus*. *Conservation Genetics Resources*, *2*(1), 101-103.
